# A parallel-risk framework accurately predicts hematopoietic stem cell transplantation outcomes and identifies benefiting patients in pediatric AML

**DOI:** 10.1016/j.gendis.2025.102003

**Published:** 2025-12-23

**Authors:** Yance Feng, Yali Shen, Ke Huang, Qian Li, Yu Tao, Rongqiu Liu, Liping Zhan, Hua Yang, Yang Xun, Yichao Xu, Wenli Tang, Binjun Xiong, Hui Shi, Liting Cheng, Li Wei, Hua You

**Affiliations:** a Precision Oncology and Intelligent Theranostics Laboratory, Department of Pediatric Hematology and Oncology Children's Hospital of Chongqing Medical University, Chongqing Key Laboratory of Pediatric Metabolism and Inflammatory Diseases, Ministry of Education Key Laboratory of Child Development and Disorders, National Clinical Research Center for Children and Adolescents' Health and Diseases, Chongqing 401122, China; b Department of Hematopoietic Stem Cell Transplantation, Children's Medical Center, Sun Yat-Sen Memorial Hospital, Sun Yat-Sen University, Guangzhou, Guangdong 510120, China; c Department of Basic Medicine, School of Medicine, Foshan University, Foshan, Guangdong 528225, China; d Chongqing Health Center for Women and Children, Women and Children's Hospital of Chongqing Medical University, NHC Key Laboratory of Birth Defects and Reproductive Health, Chongqing Municipal Health Commission Key Laboratory of Perinatal Medicine, Chongqing 401147, China

**Keywords:** Hematopoietic stem cell transplantation, Parallel-risk framework, Pediatric acute myeloid leukemia, Prognosis, Transcriptome

## Abstract

Pediatric acute myeloid leukemia (pAML) has a poorer prognosis than acute lymphoblastic leukemia, and hematopoietic stem cell transplantation (HSCT) offers curative potential in high-risk or relapsed cases. Current models cannot accurately determine which individual patients will truly benefit from HSCT, leading to overtreatment or undertreatment. We developed HSCT-64, the first parallel transcriptomic risk framework for pediatric AML, conceptually analogous to a causal G-formula approach. It comprises two treatment-specific models, aHSCT-64 for allo-HSCT recipients and nHSCT-64 for non-HSCT patients, derived from a shared 64-gene signature identified from diagnostic RNA-sequencing data, enabling individualized survival prediction under both treatment scenarios at diagnosis. Trained on 1647 cases from four COG/TARGET cohorts and validated in 233 independent patients, HSCT-64 achieved a C-index of 0.791 and AUC of 0.794 for allo-HSCT overall survival, outperforming existing clinical, cytogenetic, and leukemia stem cell-based models. Comparing risk ranks between two models identified an HSCT-benefiting subgroup patients with a predicted risk rank reduction from HSCT who experienced a 5.88-fold mortality reduction post-transplant (Hazard Ratio, HR = 0.17, *P* = 0.0066), while no survival gain was seen in the nonbenefiting subgroup (HR = 0.94, *P* = 0.899). HSCT-64 enables precise, diagnosis-time identification of pAML patients most likely to benefit from transplantation, marking a shift from high-risk-based recommendations toward individualized, transcriptome-driven decision-making.

## Introduction

Pediatric acute myeloid leukemia (pAML) accounts for approximately 15%–20% of childhood acute leukemia cases. Although its incidence is lower than that of acute lymphoblastic leukemia (ALL), its prognosis remains worse, with a 5-year survival rate of only 60%–70%. This underscores the urgent clinical need for improved, personalized treatment strategies in pediatric AML.

Hematopoietic stem cell transplantation (HSCT) represents a potentially curative approach for pAML, especially in high-risk or relapsed cases.^1^ It offers therapeutic benefit through myeloablative conditioning and the graft-versus-leukemia effect. For patients with chemoresistant disease, relapse, or high-risk genetic alterations, HSCT is often the most effective option. However, its therapeutic benefit is highly variable, and not all patients derive equal survival advantages. Therefore, an accurate, individualized prognostic model for guiding HSCT decisions is essential to optimize outcomes and avoid overtreatment.

Conventionally, HSCT is recommended for patients classified as high-risk under general models. The rationale is that these patients are unlikely to fare well with chemotherapy alone and may benefit more from aggressive interventions. Clinical trial also evidenced that HSCT was associated with improved outcomes in pediatric patients with contemporarily defined high-risk AML.^1^ To meet this requirement, risk models for AML were developed. Hematopoietic Cell Transplantation Comorbidity Index (HCT-CI)^2^ and its extensions, such as the one developed by Wang et al.,^3^ are designed to predict post-HSCT non-relapse mortality in adults but do not address pediatric contexts or pre-HSCT decision-making. The pediatric disease risk index (pDRI),^4^ which combines clinical and cytogenetic data with post-induction minimal residual disease (MRD), helps estimate transplant outcomes in pediatric AML/ALL. More recently, the pAML-SCT^5^ model incorporated basic clinical and cytogenetic features for pre-HSCT prognosis. The well-established leukemia stem cells (LSCs)-based risk model LSC17^6^^,^^7^ (for adult AML) and LSC47^8^ (for pediatric AML) represent significant advances in transcriptomic-based molecular risk stratification. Recent work advanced the risk assessment to integrate genomic, clinical, and transplantation-related factors.^9^ However, transplant outcomes vary even within the high-risk group, and it remains unclear which individual patients will truly benefit from HSCT. Notably, recent reports suggest considering HSCT for certain low-risk patients with myelodysplastic syndromes,^10^ reflecting a shift toward more nuanced decision-making.

To address this clinical challenge, we developed HSCT-64, a 64-gene transcriptomic risk model specifically tailored for pediatric AML, conceptually analogous to a causal G-formula model. This framework includes aHSCT-64 for allo-HSCT recipients and nHSCT-64 for non-HSCT patients. The models were trained and internally validated using RNA-sequencing (RNA-seq) data from diagnostic bone marrow samples of 1647 pAML cases, including 249 who received HSCT and 1398 who did not. External validation was performed in independent cohorts comprising 233 pAML patients. HSCT-64 outperformed existing transcriptomic, clinical, and cytogenetic models for both allo- and non-HSCT subgroups. By comparing patient risk ranks across the aHSCT-64 and nHSCT-64 models, we classified patients as HSCT-benefiting (risk rank decreased with transplantation) or HSCT-nonbenefiting (risk rank increased with transplantation). Transplantation significantly improved survival in the HSCT-benefiting subgroup, but conferred no advantage in the HSCT-nonbenefiting group.

In summary, HSCT-64 moves beyond the traditional high-risk-based approach to HSCT decision-making by enabling a visual and individualized assessment of transplant benefit at diagnosis, providing a precise and practical tool to support personalized treatment strategies in pediatric AML.

## Materials and methods

### Public data sources

Publicly available transcriptomic (raw counts) and clinical data from four pediatric AML cohorts (*n* = 2025) were used as the discovery cohort for prognostic gene screening and model development. These included AAML-1031 (*n* = 1082), AAML-0531 (*n* = 776), and AAML-03P1 (*n* = 100) from the Therapeutically Applicable Research to Generate Effective Treatments (TARGET) program, and the CCG-2961 cohort from the Children's Cancer Group (CCG). All datasets were accessed through GDC publicly available repositories (https://portal.gdc.cancer.gov) with appropriate usage permissions. Microarray samples of adult AML from GSE6891 (*n* = 537)^11^^,^^12^ were used to evaluate the robustness of HSCT-64 gene signature.

### Clinical cohorts and ethical approval

The general clinical cohorts (*n* = 854) used in this study comprised pediatric AML patients from two institutions: the Children's Hospital of Chongqing Medical University (CHCMU; *n* = 569) and Sun Yat-sen Memorial Hospital, Sun Yat-sen University (SYSMH; *n* = 285), collected from March 2011 to August 2023. All patients were diagnosed and treated according to institutional protocols. This study was conducted in accordance with the Declaration of Helsinki and was approved by the respective institutional review boards (IRBs) of CHCMU and SYSMH.

### RNA-sequencing

For the independent cohorts, bone marrow or peripheral blood samples were collected using anticoagulant-treated tubes, followed by RNA extraction from mononuclear cells using TRIzol kits. RNA quality was assessed by Bioanalyzer. Ribosomal RNA depletion was adopted followed by fragmentation, cDNA synthesis, adapter ligation, and PCR amplification. Sequencing was performed on Illumina NovaSeq platforms using paired-end 150 bp reads, targeting 50 million reads per sample.

### RNA-seq data analysis

For the independent cohorts, raw sequencing data were trimmed with Trim Galore, aligned to human reference genome 38 with STAR,^13^ and raw counts were quantified simultaneously with GENCODE annotation version 36. Protein-coding genes located on nuclear chromosomes were selected for normalization with CPM (count per million). The MUREN^14^ normalizer was also used for comparison and diagnosis. Principal component analysis was used to assess batch effects.

### Screening of 64 core prognostic genes

Briefly, prognostic gene screening was performed using data from 1647 pediatric AML patients in the discovery cohorts, including 249 who received allo-HSCT and 1398 who did not. The process involved: (1) initial gene filtering based on average expression, variability, and prognostic significance assessed by weighted univariate Cox regression; and (2) selection of core genes through 50 iterations of weighted elastic-net-regularized Cox modeling on 70% downsampled data, with the 64 genes most frequently selected across iterations retained as the core prognostic genes. Also, the Supplementary Methods provide a detailed discussion of the methods and their rationales.

### Development of HSCT-64

Given the 64 core prognostic genes, we transformed the survival regression problem into a series of binary classification tasks (death/survival within 1–6 years), allowing us to leverage the convenience of scikit-learn without substantial loss of statistical power. We evaluated several models, including ridge-regularized logistic regression, LASSO-regularized logistic regression, ElasticNet-regularized logistic regression (with α ranging from 0.1 to 0.9), random forest, gradient boosting, and support vector machine (SVM) classifiers. To mitigate the performance randomness introduced by data splitting, we repeated the process of training and testing across 10 random train/test splits, ensuring more robust performance evaluation. Ultimately, ridge-regularized logistic regression was selected as the final model. Using allo-HSCT cases from the discovery cohort, we developed the aHSCT-64 model, and using non-HSCT cases, we developed the nHSCT-64 model. This is equivalent to a single model where treatment type (all-HSCT or non-HSCT) interacts with each gene.

To address, or at least reduce, normalization and batch effect issues in independent RNA-seq datasets, we also constructed rank-based models (a/nHSCT-64-rank). These models were built by transforming the raw read counts of the 64 core prognostic genes into rank values (from 1 to 64), and then fitting ridge-regularized logistic regression models separately for allo-HSCT and non-HSCT cases in the discovery cohorts. These rank-based models were then applied to the independent validation data using the same rank transformation approach.

### Statistics and software

ggplot2 (https://ggplot2.tidyverse.org.)^15^ (ver 3.5.2) and ggpubr (https://github.com/kassambara/ggpubr.)^16^ (ver 0.6.0) were used for general statistical visualization. The survival^17^ (ver 3.8–3), ggsurvfit (https://github.com/ddsjoberg/ggsurvfit.)^18^ (ver 1.1.0), and survminer (https://rpkgs.datanovia.com/survminer/index.html.)^19^ (ver 0.5.0) were used for Kaplan–Meier survival curve estimation and visualization. The regularized Coxph model in the glmnet^20^ (ver 4.1–8) was used for core prognostic gene screening. Scikit-learn^21^ (ver 1.5.1) was used for model selection and fitting. C-index was calculated with glmnet. AUC was calculated with pROC^22^ (1.18.5). Differences between survival curves were tested by log-rank test.

## Results

### Patient characteristics

**Figure 1:**
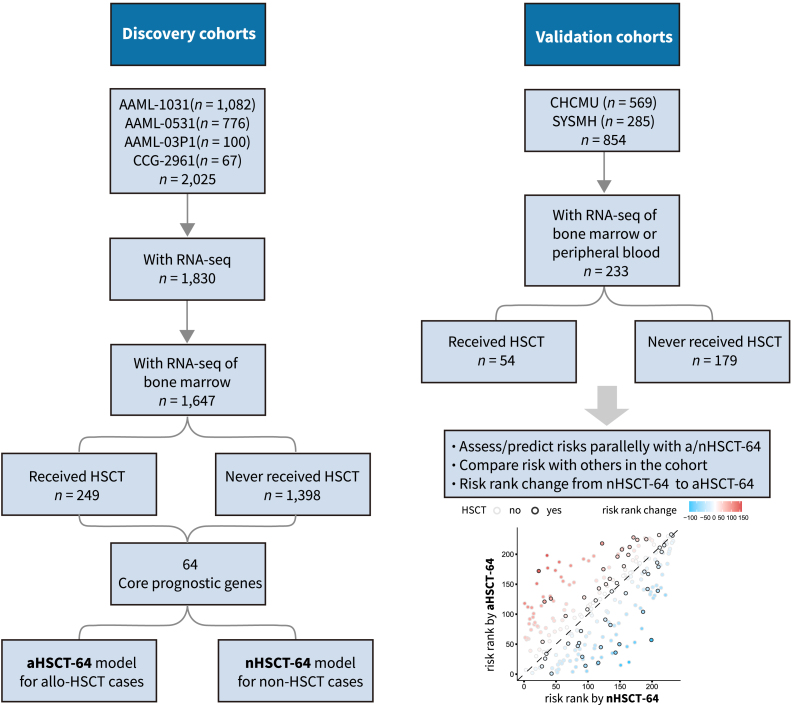
Workflow for the development, validation, and application of the HSCT-64 risk framework. A combination of four publicly available pediatric acute myeloid leukemia (pAML) cohorts was used as the discovery set to develop the HSCT-64 framework, which includes two parallel models: aHSCT-64 for allo-HSCT cases and nHSCT-64 for non-HSCT cases. Two independent pAML cohorts were employed for external validation. By comparing risk rankings generated by aHSCT-64 and nHSCT-64 in the independent cohort (*n* = 233), we identified the HSCT-benefiting subgroup (blue dots) as patients with a decreased risk rank from nHSCT-64 to aHSCT-64 and the HSCT-nonbenefiting subgroup (red dots) as those with an increased risk rank.

A combined total of four pAML cohorts (*n* = 2025) was used as discovery cohorts, including AAML-1031 (*n* = 1082),^23^, ^24^, ^25^ AAML-0531 (*n* = 776),^26^ AAML-03P1 (*n* = 100)^27^^,^^28^ from Therapeutically Applicable Research to Generate Effective Treatments (TARGET) program conducted by Children's Oncology Group (COG), as well as CCG-2961 (*n* = 67)^27^ by earlier Children's Cancer Group (CCG). Following curation, 1647 cases with RNA-seq data from diagnostic bone marrow samples were retained for core prognostic gene screening and model development, including 249 patients who received allogeneic hematopoietic stem cell transplantation (allo-HSCT) post-induction and 1398 who did not undergo HSCT. Based on a shared set of prognostic genes, two separate risk models were constructed, one for the allo-HSCT group (aHSCT-64) and one for the non-HSCT group (nHSCT-64). These two models, differing only in gene coefficients, together comprise the parallel-risk assessment framework HSCT-64 (Fig. 1).

To validate the predictive performance of the HSCT-64 model, two independent pAML cohorts (*n* = 854) were utilized: 569 cases from Children's Hospital of Chongqing Medical University (CHCMU) and 285 from Sun Yat-sen Memorial Hospital, Sun Yat-sen University (SYSMH). After data curation, 233 cases with diagnostic RNA-seq data from bone marrow or peripheral blood were retained for analysis. Key demographic and clinical characteristics are summarized in Table 1.

**Table 1:** Clinical summary of discovery and validation cohorts.

Features	Discovery cohorts (*n* = 1647)		Validation Cohorts (*n* = 223)	
	Non-HSCT cases	Allo-HSCT cases	Non-HSCT cases	Allo-HSCT cases
Cases (%)	1398 (84.9)	249 (15.1)	179 (76.8)	54 (23.2)
Age in years (90% CI^a^)	10.2 (0.8, 18.8)	10.4 (1.3, 18.9)	7.3 (1.2, 14.2)	4.6 (1.2, 14.0)
Gender (male%)	51.3	57.4	53.6	58.3
Race (%)	White (79.9)	White (85.2)	Chinese (100)	Chinese (100)
WBC^b^, × 10^9^/L (90% CI)	25.5 (2.4, 214.1)	25.9 (1.6, 262.7)	12.8 (1.6, 129.8)	11.7 (1.6, 112.9)
PB blasts%^c^ (90% CI)	41.0 (0.0, 91.0)	42.0 (0.0, 93.3)	39.0 (2.5, 89.0)	42.0 (8.4, 73.6)
BM blasts%^d^ (90% CI)	69.6 (21.0, 95.0)	75.0 (24.1, 95.0)	73.3 (19.8, 96.4)	60.5 (22.5, 95.7)
Median follow-up^e^ (years)	4.8	4.6	2.8	2.1

a CI, confidence interval.

b WBC, white blood cell counts at diagnosis.

c PB Blasts, peripheral blood blasts at diagnosis.

d BM Blasts, bone marrow blasts at diagnosis.

e Estimated by reverse-status Kaplan–Meier (KM) method.

### Screening of core prognostic genes and development of parallel risk framework HSCT-64

A critical step in constructing transcriptional (omics)-based risk models is the identification of core prognostic genes. To this end, we employed a robust, data-driven approach (see Methods). Following appropriate preprocessing, batch effects across cohorts and related confounding factors were effectively corrected and verified (Fig. S1). We first applied weighted univariate Cox proportional hazards models to identify genes significantly associated with overall survival (OS) (Fig. S2A; Table S1). The top 1000 genes from this step were then subjected to multivariate analysis using a weighted elastic net-regularized Cox proportional hazards model. To ensure robustness, we performed 50 subsampling iterations, in each of which 70% of the samples were randomly selected for model training and 30% for testing. The most frequent 64 genes among the 50 iterations were retained as the final list of core prognostic genes (Table S2). Gene Ontology analysis indicated that these genes are biologically meaningful (Table S3 and S4) and notably enriched in fatty acid metabolism pathways (Fig. S2B–S2D). To demonstrate the robustness of the HSCT-64 gene signature, we compared the expression patterns across RNA-seq samples used in this study with microarray data of 537 adult AML patients.^11^^,^^12^ The expression quantiles in the RNA-seq samples were highly concordant with those in the microarray data, with a median correlation of 0.983 (Fig. S3).

**Figure 2:**
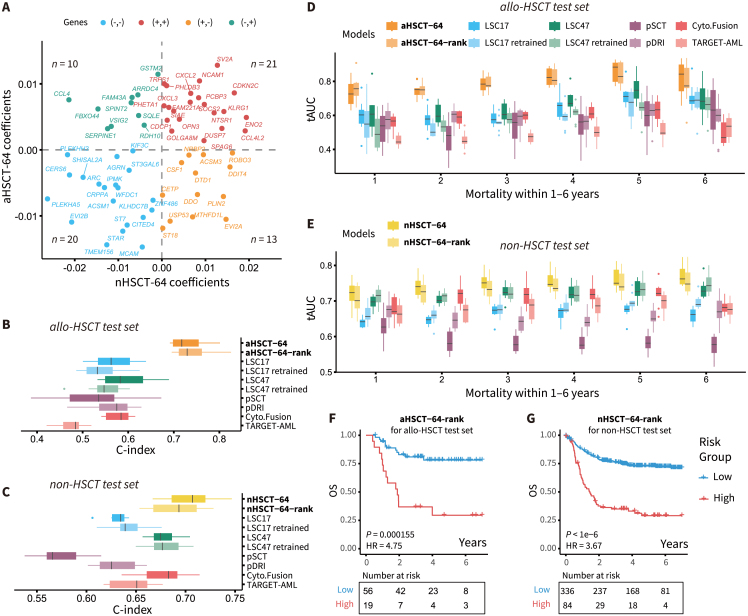
Development of the HSCT-64 model and its performance on internal test sets from the discovery cohorts. **(A)** Model coefficients for aHSCT-64 and nHSCT-64. The 64 core prognostic genes were classified into four categories based on their associations with risk: for example, the expression levels of 21 genes (red) were positively associated with increased mortality risk in both models; the remaining three categories follow similar interpretations. **(B, C)** Allo-HSCT (B) and non-HSCT (C) cases from the discovery cohorts were randomly split into training (70%) and testing (30%) sets. The aHSCT-64 and nHSCT-64 models were trained on the training sets, and their predictive performance was evaluated on the test sets using the concordance index (C-index) with respect to overall survival (OS). **(D, E)** As in (B, C), but evaluated using the time-dependent area under the ROC curve (tAUC) for predicting death/alive within 1–6 years respectively. **(F, G)** Kaplan–Meier survival analysis of high-risk and low-risk subgroups defined by aHSCT-64-rank (F) and nHSCT-64-rank (G) in the respective test sets. Significant survival differences were observed (log-rank test). HR: hazard ratio.

Following gene selection, we reframed the task as a classification problem to predict death within 1–6 years and evaluated a range of machine learning models, both linear and non-linear (Fig. S2E and S2F). Ultimately, we selected a ridge-regularized logistic regression model for its balance of performance and interpretability. This model was trained separately on allo-HSCT (aHSCT-64) and non-HSCT (nHSCT-64) cases in the discovery cohort. Although both models shared the same 64 genes, their coefficients differed, reflecting the differential prognostic impact of HSCT (Fig. 2A; Table S5).

To mitigate the impact of random training/test splits on model performance, we repeated the entire process 10 times using different random seeds. In each iteration, the dataset was split into training and test sets, a/nHSCT-64 models were trained, and performance was evaluated using the test sets. We assessed concordance index (C-index) for OS and time-dependent AUCs for predicting death within 1–6 years. The aHSCT-64 (Fig. 2B–D) and nHSCT-64 (Fig. 2C–E) models achieved median C-index and AUC values that exceeded those of several benchmark models, including pediatric disease risk indices based on clinical and cytogenetic features (pDRI,^3^ pSCT^5^), gene fusion-based stratification (CytoFusion), and the TARGET-AML defined risk categories in allo-HSCT (Fig. S4 and S5) and non-HSCT cases (Fig. S6 and S7), respectively. As no transcriptomic-based HSCT risk models for pAML have been previously published, we also compared performance against two leukemia stem cell-based models: LSC17^6^^,^^7^ (developed for adult AML) and LSC47^8^ (developed for pediatric AML), including versions retrained on our training data. Based on a/nHSCT-64 model risk scores, we stratified the test sets into high- and low-risk groups. Kaplan–Meier analysis revealed significantly different OS between these groups (Fig. 2F and G), highlighting the strong stratification ability of a/nHSCT-64. Similar results were observed when evaluating event-free survival (EFS) across the models (Fig. S5 and S7). Moreover, the HSCT-64 models demonstrated robust predictive performance across sample types, with slightly higher concordance in bone marrow than peripheral blood (overall C-index ≈ 0.70–0.78, Table S6).

Additionally, to address the challenges of normalization and batch effects in transcriptomic data, we also developed a rank-based variant of the model (Fig. S1). Raw expression counts of the 64 prognostic genes were transformed into within-sample ranks (1–64), and rank-based models (aHSCT-64-rank and nHSCT-64-rank) were trained accordingly. These models achieved comparable performance to the original versions (Fig. 2B–E), further supporting the robustness of the HSCT-64 framework. See Supplementary Methods for further discussion on methodological rationale.

### HSCT-64 accurately predicts HSCT and non-HSCT survival in independent cohorts

To evaluate the predictive performance of the HSCT-64 model on independent pediatric AML cohorts, we applied the robust, rank-based a/nHSCT-64 models to a combined set of 233 diagnostic RNA-seq samples from CHCMU and SYSMH. This cohorts included 54 patients who underwent allo-HSCT and 179 who did not. The models operated on within-sample ranks of raw RNA-seq counts (Table S4, Table S6) for the 64 core prognostic genes. Crucially, no additional tuning or modifications were made to the models, and the independent data were not selectively filtered, ensuring an unbiased and transparent evaluation.

**Figure 3:**
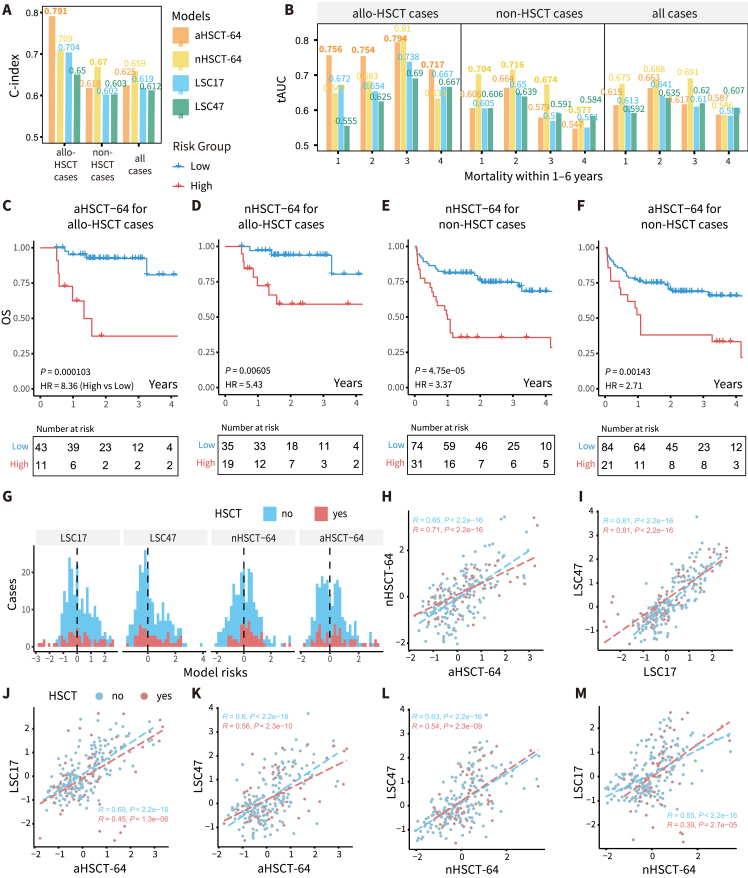
Validation of HSCT-64 performance in independent cohorts. **(A, B)** Comparison of C-index (A) and time-dependent AUC (tAUC) (B) for overall survival (OS) across different models in allo-HSCT, non-HSCT, and combined cases from independent cohorts. **(C, D)** Kaplan–Meier survival analysis of high- and low-risk subgroups defined by aHSCT-64 (C) and nHSCT-64 (D) in allo-HSCT cases. **(E, F)** Kaplan–Meier survival analysis of high- and low-risk subgroups defined by aHSCT-64 (E) and nHSCT-64 (F) in non-HSCT cases. **(G)** Distribution of scaled risk scores (zero median, unit standard deviation) predicted by each model for allo-HSCT (red) and non-HSCT (blue) cases in the independent cohorts. **(H**–**M)** Pairwise risk comparisons among aHSCT-64, nHSCT-64, LSC17, and LSC47 models. Pearson's correlation coefficients (R) between model-predicted risks are shown for allo-HSCT (red) and non-HSCT (blue) cases. Correlations were assessed using Fisher's *Z* test.

For OS prediction, the aHSCT-64 and nHSCT-64 models achieved C-indices of 0.791 and 0.670, respectively, both outperforming the LSC-based models LSC17 and LSC47 (Fig. 3A). In time-dependent ROC analysis for predicting death events within 1–4 years, the AUCs peaked at 0.794 for aHSCT-64 and 0.716 for nHSCT-64 (Fig. 3B). For sensitivity comparison, we also evaluated model performance on patients outside their respective target groups, as well as on the full cohort (Fig. 3A and B). Risk stratification by aHSCT-64 significantly separated allo-HSCT patients into groups with distinct OS outcomes (log-rank *P* = 1.03e-4, HR = 8.36; Fig. 3C). Similarly, nHSCT-64 stratified non-HSCT patients into groups with significantly different survival outcomes (log-rank *P* = 4.75e-5, HR = 3.37; Fig. 3E), outperforming LSC17 and LSC47 (Fig. S8A–S8D). Although still effective, both models showed slightly diminished stratification capacity when applied to non-target groups (Fig. 3D–F). Similar results for EFS were also evaluated (Fig. S8E–S8N).

To further contrast HSCT-64 with LSC-based models, we compared the marginal (Fig. 3G) and joint (Fig. 3H–M) distributions of standardized risk scores (zero median, unit standard deviation) predicted by a/nHSCT-64 (Table S5), LSC17, and LSC47. For allo-HSCT and non-HSCT cases, aHSCT-64 and nHSCT-64 risks were moderately correlated, with Pearson's *R* values of 0.71 and 0.64, respectively (Fig. 3H). In contrast, LSC17 and LSC47 showed stronger correlations (*R* = 0.81) in both groups (Fig. 3I). Cross-model correlations between a/nHSCT-64 and LSC17/LSC47 were moderate: *R* = 0.39–0.56 for allo-HSCT cases and *R* = 0.55–0.69 for non-HSCT cases (Fig. 3J–M), suggesting that a/nHSCT-64 captures distinct risk profiles. In summary, the aHSCT-64 and nHSCT-64 models provide more accurate and treatment-specific risk predictions for pediatric AML patients undergoing or not undergoing HSCT.

### HSCT-64 identifies HSCT-benefiting patients at diagnosis

**Figure 4:**
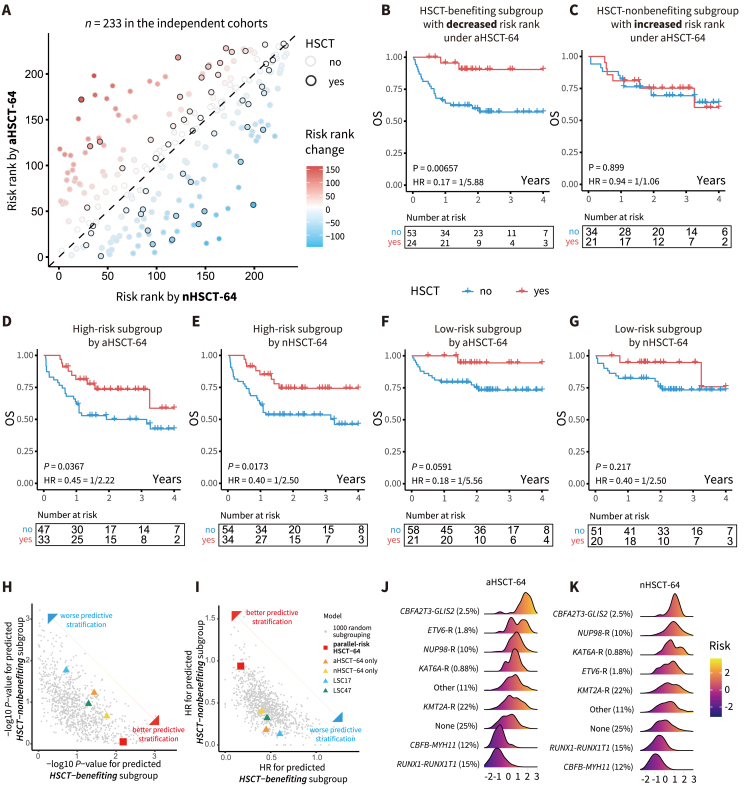
Identification of HSCT-benefiting patients using the HSCT-64 parallel-risk framework at diagnosis. **(A)** Comparison of risk ranks generated by aHSCT-64 and nHSCT-64 models in the independent cohort. Patients with a decreased risk rank under aHSCT-64 (blue) were predicted to benefit from HSCT, while those with an increased rank (red) were predicted as non-benefiting. **(B)** Among the predicted HSCT-benefiting subgroup, transplanted patients showed significantly better overall survival (OS) than non-transplanted patients. **(C)** In the HSCT-nonbenefiting subgroup, transplantation did not lead to a significant difference in OS. **(D, E)** Kaplan–Meier survival analysis comparing transplanted and non-transplanted patients in the high-risk subgroup defined by aHSCT-64 (D) and nHSCT-64 (E). **(F, G)** Kaplan–Meier survival analysis comparing transplanted and non-transplanted patients in the low-risk subgroup defined by aHSCT-64 (F) and nHSCT-64 (G). **(H, I)** Distributions of *p*-values (H) and HRs (I) among 1000-replicate bootstrap predicting cases as HSCT-benefiting and HSCT-nonbenefiting illustrated that the parallel-risk HSCT framework had more predictive stratification. **(J, K)** Risk distributions of genetic (gene fusion) subtypes assessed by aHSCT-64 (J) and nHSCT-64 (K), with subtypes ordered according to median risk. Differences in survival curves were evaluated using the log-rank test. HR: hazard ratio.

Equipped with the a/nHSCT-64 models, we can estimate each patient's survival risk under two parallel scenarios: one assuming the patient receives allo-HSCT, and the other assuming they do not. By comparing the risk ranks within the entire independent cohort (Fig. 4A; Table S5), we define a patient as likely to benefit from HSCT if their predicted risk rank decreases (blue points) from nHSCT-64 to aHSCT-64. Conversely, an increase in risk rank (red points) suggests that HSCT may not be beneficial. Notably, in the HSCT-benefiting subgroup (defined by a risk rank reduction of greater than 10), the transplanted patients showed significantly better OS than their non-transplanted counterparts (log-rank *P* = 0.00657; HR = 0.17), corresponding to a 5.88-fold reduction in mortality risk (Fig. 4B). In contrast, for the HSCT-nonbenefiting subgroup (defined by a risk rank increase greater than 10), no significant survival advantage was observed for HSCT (*P* = 0.899; HR = 0.94; Fig. 4C).

Traditionally, risk stratification relies on a single model, typically applied to all patients or exclusively to those receiving HSCT, where high-risk patients are recommended for more aggressive treatments like transplantation. This strategy assumes that high-risk patients fare poorly with standard therapy and may benefit from HSCT, but it does not assess whether HSCT is actually advantageous for each individual. For example, using aHSCT-64 alone to stratify risk, transplanted patients in the high-risk group did show improved survival compared to non-transplanted patients (*P* = 0.0367; HR = 0.45; Fig. 4D), but the effect was notably weaker than that observed in the HSCT-benefiting group identified by the parallel-risk framework (Fig. 4B). Furthermore, among low-risk patients as defined by aHSCT-64, there was a trend toward better OS in transplanted patients, although not statistically significant (Fig. 4E). Similar results were observed when using nHSCT-64 for stratification (Fig. 4F and G), LSC17 (Fig. S9A and S9B), and LSC47 (Fig. S9C and S9D). Moreover, using risk score change instead of risk rank change for subgrouping, HSCT-64 remained effective (Fig. S10).

To demonstrate the greater discriminatory power of the parallel-risk HSCT-64 framework, we compared it with randomly assigned HSCT-benefiting and HSCT-nonbenefiting subgroups. Within each subgroup, we evaluated OS differences between allo-HSCT and non-HSCT patients using Cox proportional hazards models. The bootstrap distributions (Table S7) of *P*-values (Fig. 4H) and hazard ratios (Fig. 4I) confirmed that the parallel-risk framework more reliably identifies patients who are truly likely to benefit from transplantation.

In addition, patients harboring different genetic mutations or gene fusions exhibited distinct risk distributions when assessed by both aHSCT-64 (Fig. 4J) and nHSCT-64 (Fig. 4K). When ordered by median risk, these genetic subgroups showed a pattern consistent with their known clinical outcomes.^29^ In contrast, patients stratified by the 2022 ELN classification, developed primarily based on adult AML cohorts, displayed largely overlapping HSCT-64 risk distributions (Fig. S11A and S11B) and similar clinical outcomes (Fig. S11C and S11D), underscoring its limited applicability to pediatric AML.

In summary, by evaluating each patient's risk in two hypothetical treatment scenarios using the a/nHSCT-64 models, we can make individualized predictions regarding the likely effect of transplantation. This parallel-risk approach enables the identification of HSCT-benefiting and HSCT-nonbenefiting subgroups at diagnosis, offering more precise and clinically actionable guidance than traditional risk stratification strategies that recommend HSCT for all high-risk patients.

## Discussion

In terms of precision medicine, we have a clear answer to the question of whether HSCT should be broadly recommended for all high-risk patients or selectively prioritized for those most likely to benefit: it should be the latter. However, since each patient either undergoes HSCT or not, it is inherently difficult to estimate the individual-level benefit of transplantation on survival. Until now, no existing model has been able to identify a subgroup of patients who would truly benefit from HSCT.

HSCT-64 differs fundamentally from conventional prognostic models that merely predict relapse or survival risk. By modeling the parallel risks under HSCT and non-HSCT scenarios (analogous to the G-formula in causal inference), HSCT-64 identifies pediatric AML patients who truly benefit from transplantation. This approach challenges the traditional ‘high-risk-requires-HSCT’ concept and supports individualized, benefit-based transplant decision-making.

Current adult AML prognostic models are not directly applicable to pediatric patients due to key differences in epidemiology, genetics, and therapeutic response. Adult AML primarily affects older individuals and presents with a more complex mutational landscape, including frequent alterations in *FLT3*-ITD, *NPM1*, *IDH1/2*, and *DNMT3A*. In contrast, pediatric AML more commonly involves gene fusions such as *KMT2A* rearrangements, *RUNX1*-*RUNX1T1*, and *CBFB*-*MYH11*. Pediatric patients also tend to respond better to chemotherapy, and many standard-risk cases can be cured without HSCT. Moreover, our previous work and other studies have shown that adult models, such as ELN-2022 and the leukemia stem cell-based LSC17, perform suboptimally in pediatric AML.^30^^,^^31^ Hence, it is indispensable to develop tailored risk models for the treatment decision of pediatric AML patients.

Clinically, factors influencing HSCT outcomes include patient-related variables (*e.g.*, age, sex), disease-related characteristics (*e.g.*, cytogenetic and molecular abnormalities), donor and transplant type, as well as transplant-related complications such as graft-versus-host disease (GVHD). Although HSCT-64 relies solely on the expression levels of 64 genes at diagnosis and does not incorporate these clinical variables directly, it still achieves high predictive accuracy for HSCT outcomes. This is likely because many of these clinical and biological factors are inherently reflected in the global gene expression profile. Rather than modeling each factor individually, HSCT-64 captures their combined effects through a direct association between transcriptomic signatures and post-transplant survival, bypassing the need for intermediate clinical inputs. Moreover, relying solely on RNA-seq-based gene expression for prognosis helps overcome biases introduced by MRD testing platforms and minimizes subjectivity in clinical assessment.

The HSCT-64 model encompasses genes involved in fatty-acid metabolism, lipid regulation, and immune modulation, suggesting a metabolic–immune axis underlying post-transplant prognosis. Several key members, such as *ACSM1* and *ACSM3*, catalyze fatty-acid activation and support β-oxidation, while *PLIN2*, *SQLE*, and *CERS6* regulate lipid droplet formation, sterol synthesis, and ceramide homeostasis, processes known to sustain leukemia stem-cell quiescence and resistance to oxidative stress.^32^^,^^33^ Elevated expression of these genes may endow leukemic cells with metabolic flexibility that enables persistence after conditioning and contributes to relapse following HSCT. In parallel, immune-related genes including *SOCS2*, *CXCL2*, *CXCL3*, and *SERPINE1* participate in cytokine signaling and inflammatory regulation, potentially shaping the graft-versus-leukemia and graft-versus-host disease (GVHD) balance.^34^^,^^35^ Together, these metabolic and immune signatures may delineate a leukemia state less responsive to cytotoxic or immune-mediated clearance. Clinically, the HSCT-64 profile highlights metabolic vulnerabilities that could be exploited therapeutically, such as inhibition of fatty-acid oxidation or sterol biosynthesis, to enhance transplant efficacy and prevent relapse.^36^^,^^37^

Despite these promising findings, several limitations should be acknowledged. Beyond the moderate sample size of the validation cohort, potential sources of bias include single-center recruitment, regional and ethnic homogeneity, and a relatively short follow-up duration compared with the discovery cohorts. Nevertheless, the heterogeneity among cohorts in sample source, ethnicity, and clinical characteristics may also serve to validate the clinical generalizability of the HSCT-64 model. Additionally, while transcriptomic data provide high-resolution insights into gene expression states, integrating other omics layers, such as genomic, epigenomic, and proteomic data, could further refine biological interpretation and improve model robustness. Future multi-omics and multicenter validations will be critical to confirm the reproducibility and translational potential of HSCT-64 in broader clinical settings.

## CRediT authorship contribution statement

**Yance Feng:** Writing – review & editing, Writing – original draft, Visualization, Validation, Methodology, Investigation, Formal analysis, Data curation, Conceptualization. **Yali Shen:** Resources, Investigation, Funding acquisition. **Ke Huang:** Resources. **Qian Li:** Formal analysis. **Yu Tao:** Formal analysis, Data curation. **Rongqiu Liu:** Formal analysis, Data curation. **Liping Zhan:** Data curation. **Hua Yang:** Data curation. **Yang Xun:** Data curation. **Yichao Xu:** Formal analysis. **Wenli Tang:** Formal analysis. **Binjun Xiong:** Formal analysis. **Hui Shi:** Data curation. **Liting Cheng:** Formal analysis. **Li Wei:** Funding acquisition, Conceptualization. **Hua You:** Writing – original draft, Supervision, Resources, Project administration, Investigation, Funding acquisition, Conceptualization.

## Ethics declaration

This study was conducted in accordance with the Declaration of Helsinki and was approved by the respective institutional review boards (IRBs) of CHCMU (Approval No. 2023-454) and SYSMH (Approval No. SYSKY-2025-621-01).

## Data availability

Publicly available RNA-seq and clinical data from four pediatric AML cohorts (AAML-1031, AAML-0531, AAML-03P1, and CCG-2961) were obtained from the NCI Genomic Data Commons (GDC) data portal under appropriate usage permissions. Microarray-based gene expression data of adult AML patients were obtained from the GEO database (accession GSE6891). Independent validation datasets generated at the Children's Hospital of Chongqing Medical University (CHCMU) and Sun Yat-sen Memorial Hospital (SYSMH) are not publicly available due to patient privacy and institutional regulations, but de-identified data supporting the findings of this study are available from the corresponding author upon reasonable request and subject to institutional approvals.

## Funding

The research activities were mainly supported by the 10.13039/501100001809National Natural Science Foundation of China (No. 81911530169), Joint Project of Chongqing Health Commission and Science and Technology Bureau (China) (No. 2025ZDXM005, 2026MSXM022, 2026QNXM029), CQMU Program for Youth Innovation in Future Medicine (China) (No. W0202), the Science and Technology Research Program of Chongqing Municipal Education Commission (China) (No. KJZD K202300408), the Innovation Support Program for Chongqing Overseas Returnees (cx2025115), 10.13039/501100002865Chongqing Science and Technology Bureau (Grant No.CSTB2025NSCQ-GPX0396), and the First-Class Discipline Development Program in Clinical Medicine of Children's Hospital of Chongqing Medical University (China) (No. CHCMU-2025-YLXK-008).

## Conflict of interests

The authors declare no competing interests.
