# Supplementary material for: A parallel-risk framework accurately predicts hematopoietic stem cell transplantation outcomes and identifies benefiting patients in pediatric AML
Source: Genes Dis. 2025 Dec 23;13(5):102003. doi: 10.1016/j.gendis.2025.102003 (PMC13273867; doi:10.1016/j.gendis.2025.102003)
Supplement: Multimedia component 1 [file mmc1.docx]

# **Supplementary Figures**

**Figure S1**. Principal component analysis (PCA) for batch effect assessment.

**Figure S2**. Screening of core prognostic genes and evaluation of statistical learning models.

**Figure S3**. Cross-platform evaluation of the robustness of HSCT-64 gene expression between RNA-seq and microarray datasets.

**Figure S4**. Evaluation of risk models for overall survival (OS) on the discovery allo-HSCT test set.

**Figure S5**. Evaluation of risk models for event-free survival (EFS) on the discovery allo-HSCT test set.

**Figure S6**. Evaluation of risk models for overall survival (OS) on the discovery non-HSCT test set.

**Figure S7**. Evaluation of risk models for event-free survival (EFS) on the discovery non-HSCT test set.

**Figure S8**. Validation of HSCT-64 performance in independent cohorts.

**Figure S9**. HSCT decision by single risk model.

**Figure S10**. HSCT decision by parallel-risk model HSCT-64.

**Figure S11**. HSCT-64 risk distribution and clinical outcomes according to the 2022 European Leukemia Net (ELN) risk classification at initial diagnosis.


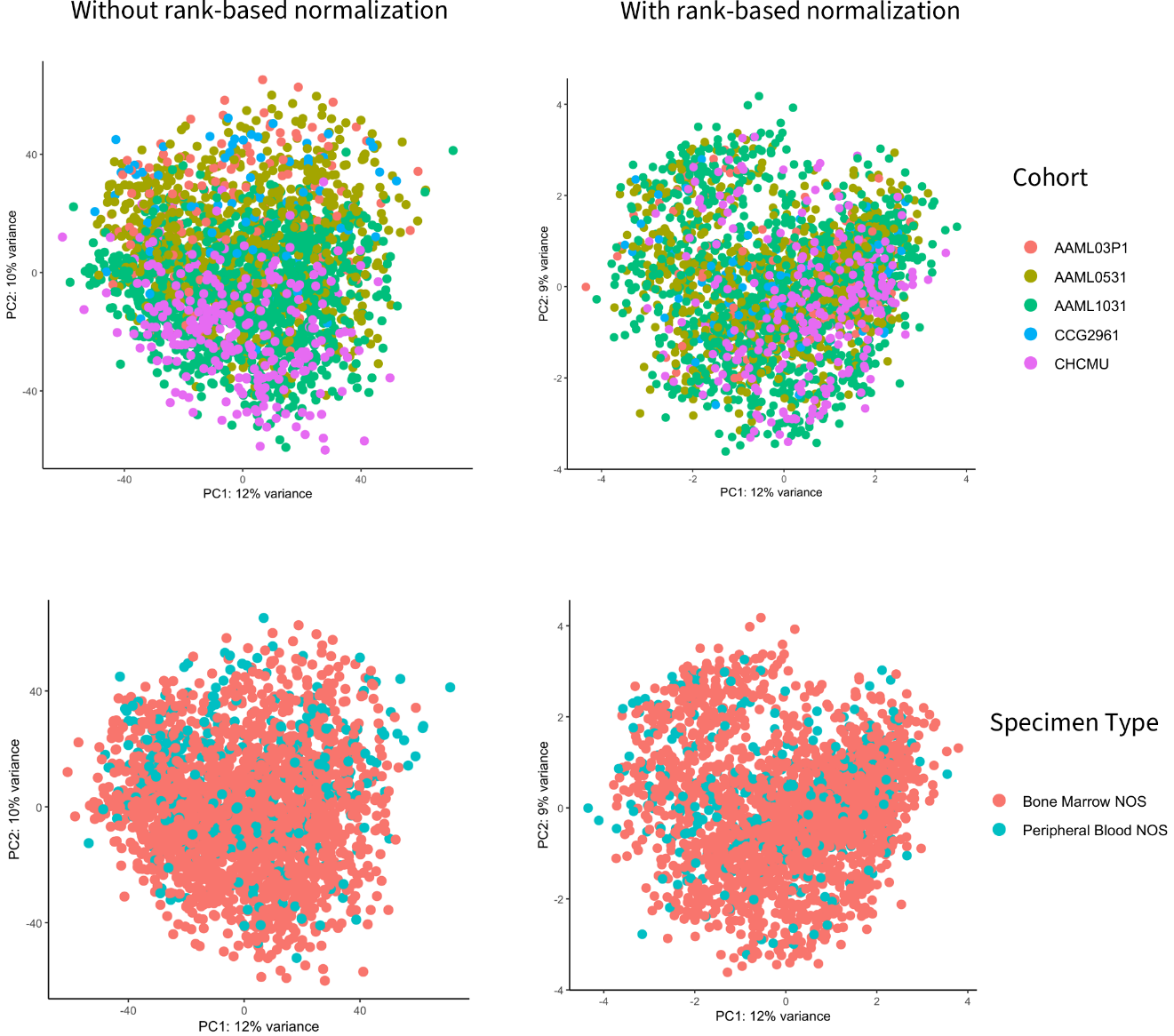


**Figure S1. Principal component analysis (PCA) for batch effect assessment.**

After rank-based normalization, batch effects among cohorts were effectively corrected, and no apparent separation was observed between bone marrow and peripheral blood samples, as shown by the first two principal components using the top 1,000 highly variable genes.


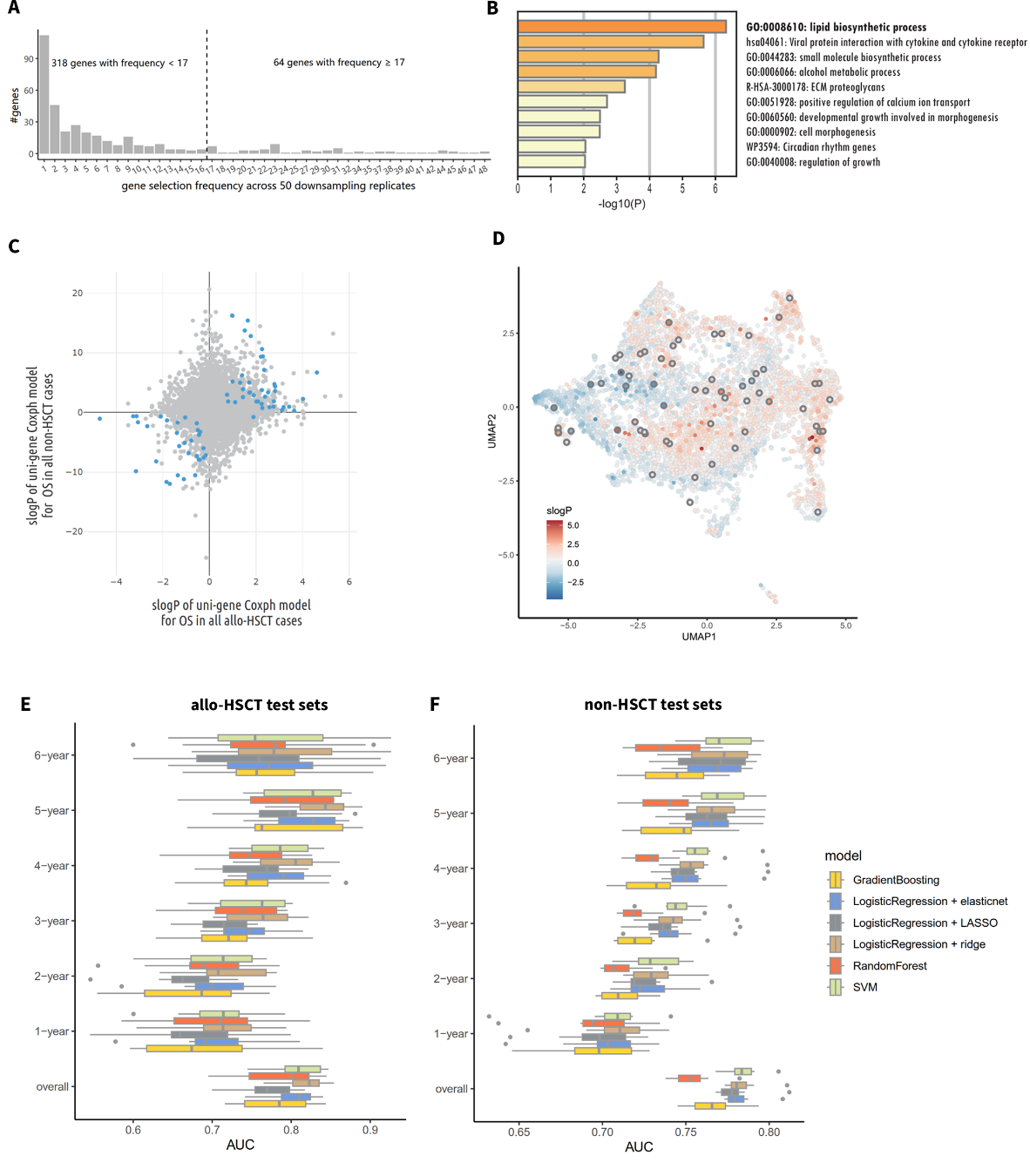


**Figure S2. Screening of core prognostic genes and evaluation of statistical learning models.**

(**A**) Core prognostic genes were identified through 50 repeated downsampling screenings using a weighted elasticnet-regularized Cox proportional hazards model. A total of 64 genes selected in ≥17 iterations were retained as core prognostic genes. (**B**) Gene Ontology enrichment analysis of the 64 core genes revealed significant functional associations. (**C**) Signed log10 P-values (slogP) from univariate Cox models for overall survival in allo-HSCT cases (x-axis) and non-HSCT cases (y-axis), showing gene-specific prognostic relevance across conditions. (**D**) UMAP projection of 7,000 candidate prognostic genes based on expression profiles in all discovery samples. The 64 core genes are highlighted in gray. (**E–F**) Predictive performance of various statistical learning models trained on the 64 core genes was evaluated for death/survival classification at 1–6 years. AUC distributions are shown for allo-HSCT (E) and non-HSCT (F) cohorts, based on 10 repeated rounds of data splitting, model training, and testing to reduce random bias. Elasticnet-regularized logistic regression mode outperformed other models and was used as final model.


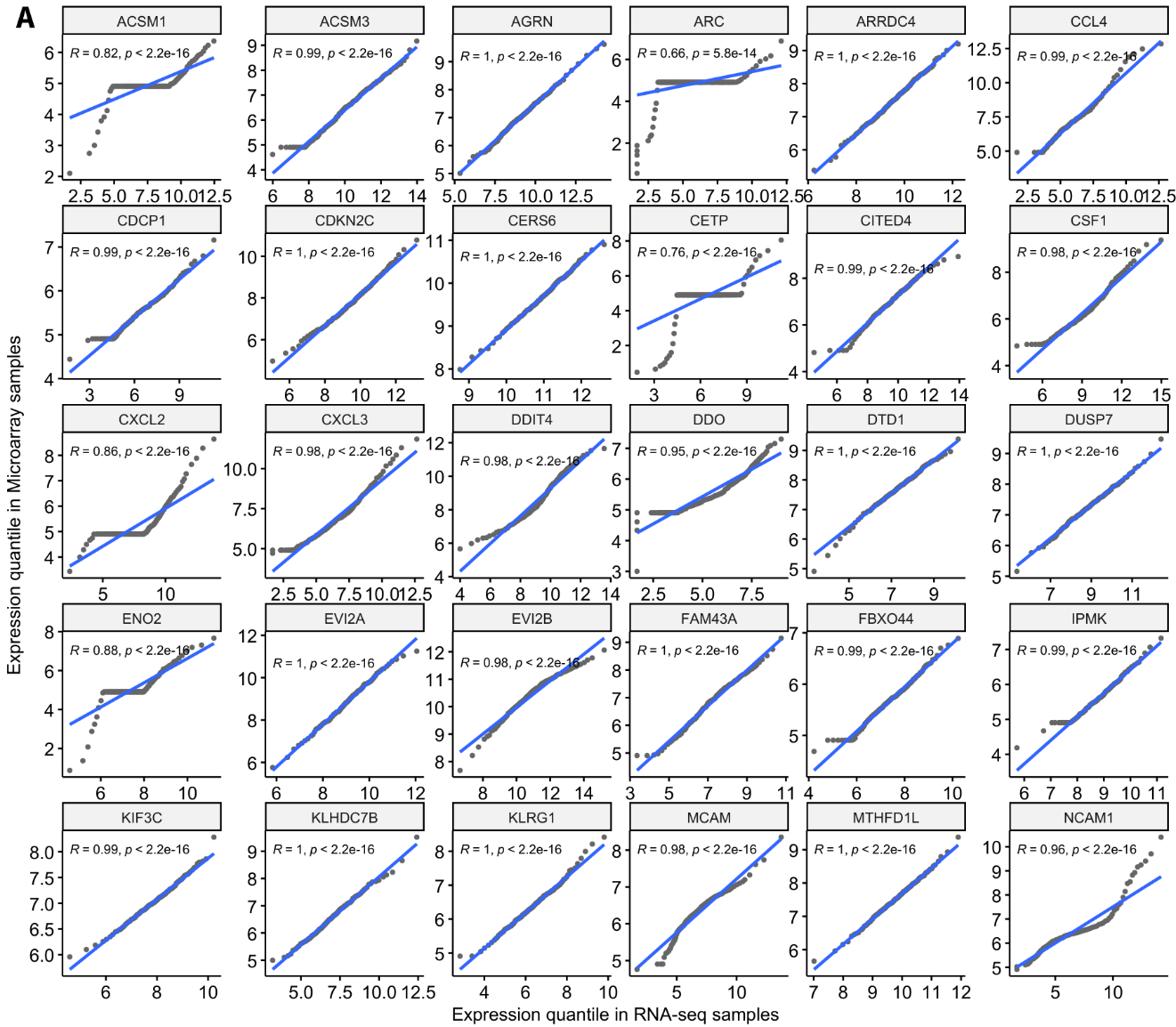


**Figure S3. Cross-platform evaluation of HSCT-64 gene expression robustness between RNA-seq and microarray data. (To be continued)**


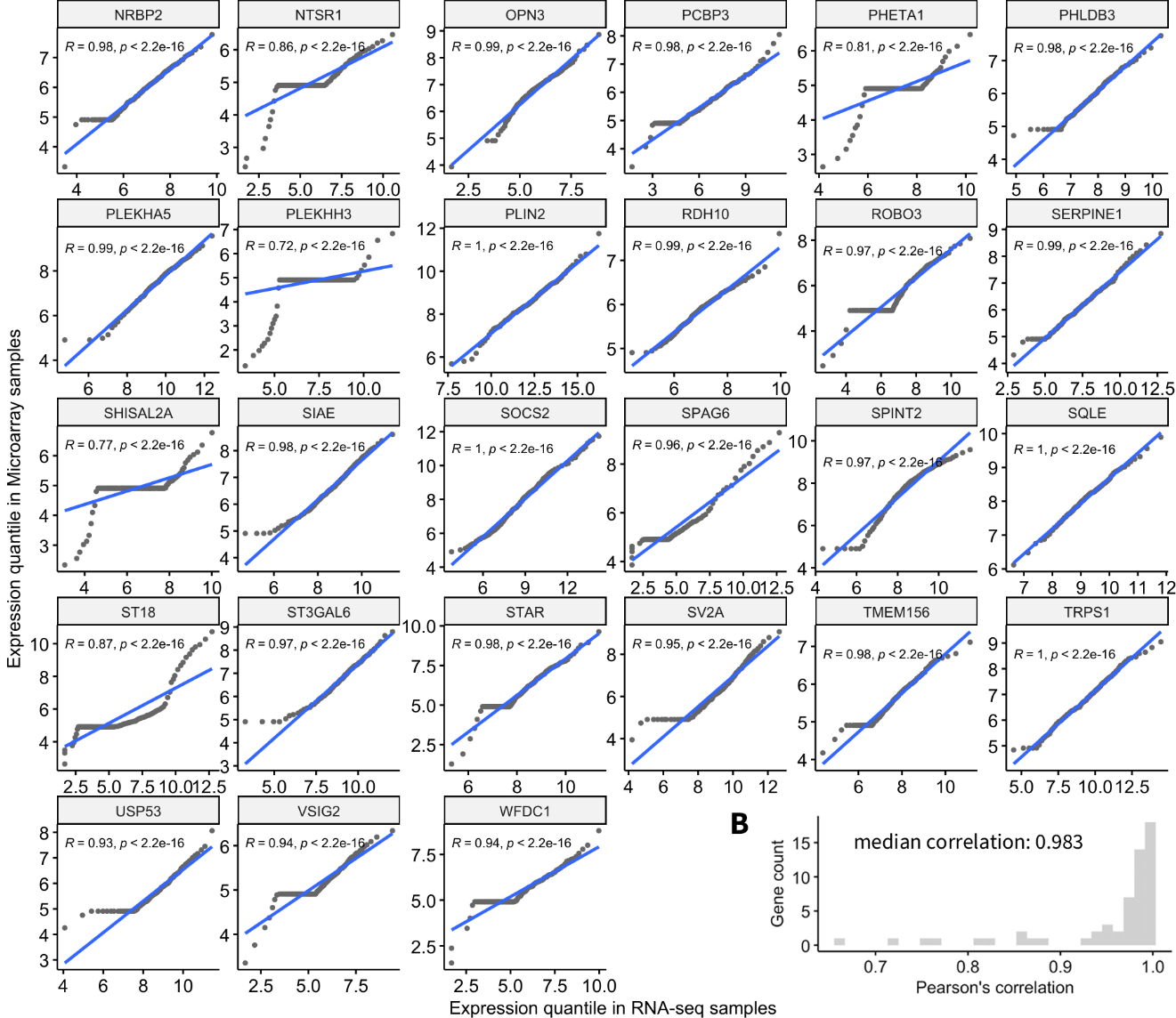


***Figure S3 (continued). Cross-platform evaluation of the robustness of HSCT-64 gene expression between RNA-seq and microarray datasets.***

*(****A****) Quantile–quantile plots comparing RNA-seq samples from this study with microarray samples (Affymetrix Human Genome U133 Plus 2.0 Array) from GSE6891 (n = 537) for each HSCT-64 gene. Robust regression lines (blue) were fitted using M-estimation (MASS::rlm() in R). (****B****) Distribution of Pearson correlation coefficients between RNA-seq and microarray quantiles, with a median correlation of 0.983, demonstrating high cross-platform consistency.*
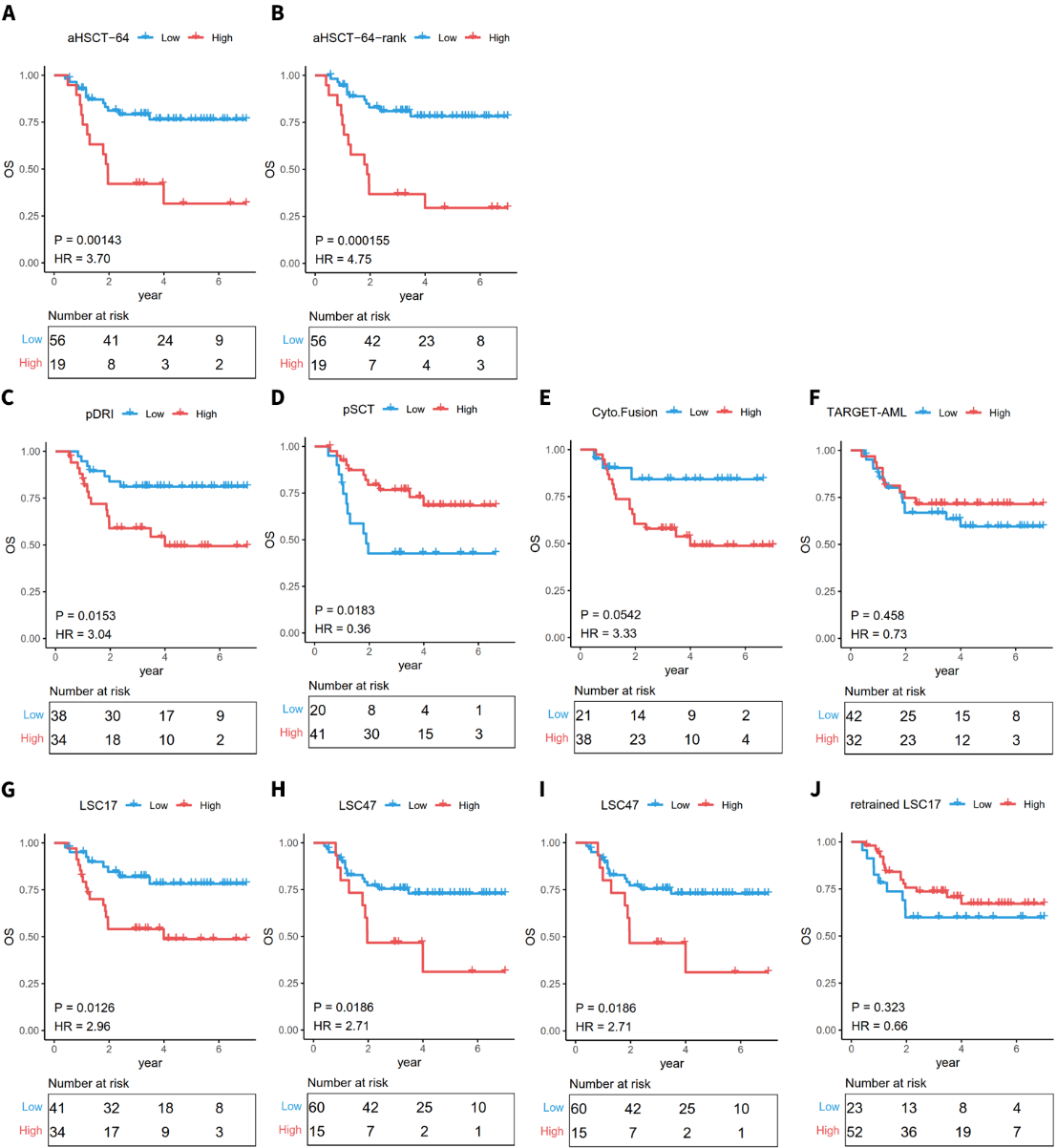


**Figure S4. Evaluation of risk models for overall survival (OS) on the discovery allo-HSCT test set.**

(**A-J**) Kaplan–Meier analysis comparing EFS between low- and high-risk subgroups stratified by aHSCT-64 (A), rank-based aHSCT-64-rank (B), pDRI (C), pSCT (D), cytogenetic gene fusion-based Cyto.Fusion (E), TARGET-AML risk group (F), leukemia stem cells (LSCs)-based risk model LSC17 (G), LSC17 retained on training sets (H), LSC47 (I), and LSC47 retained on training sets (J).


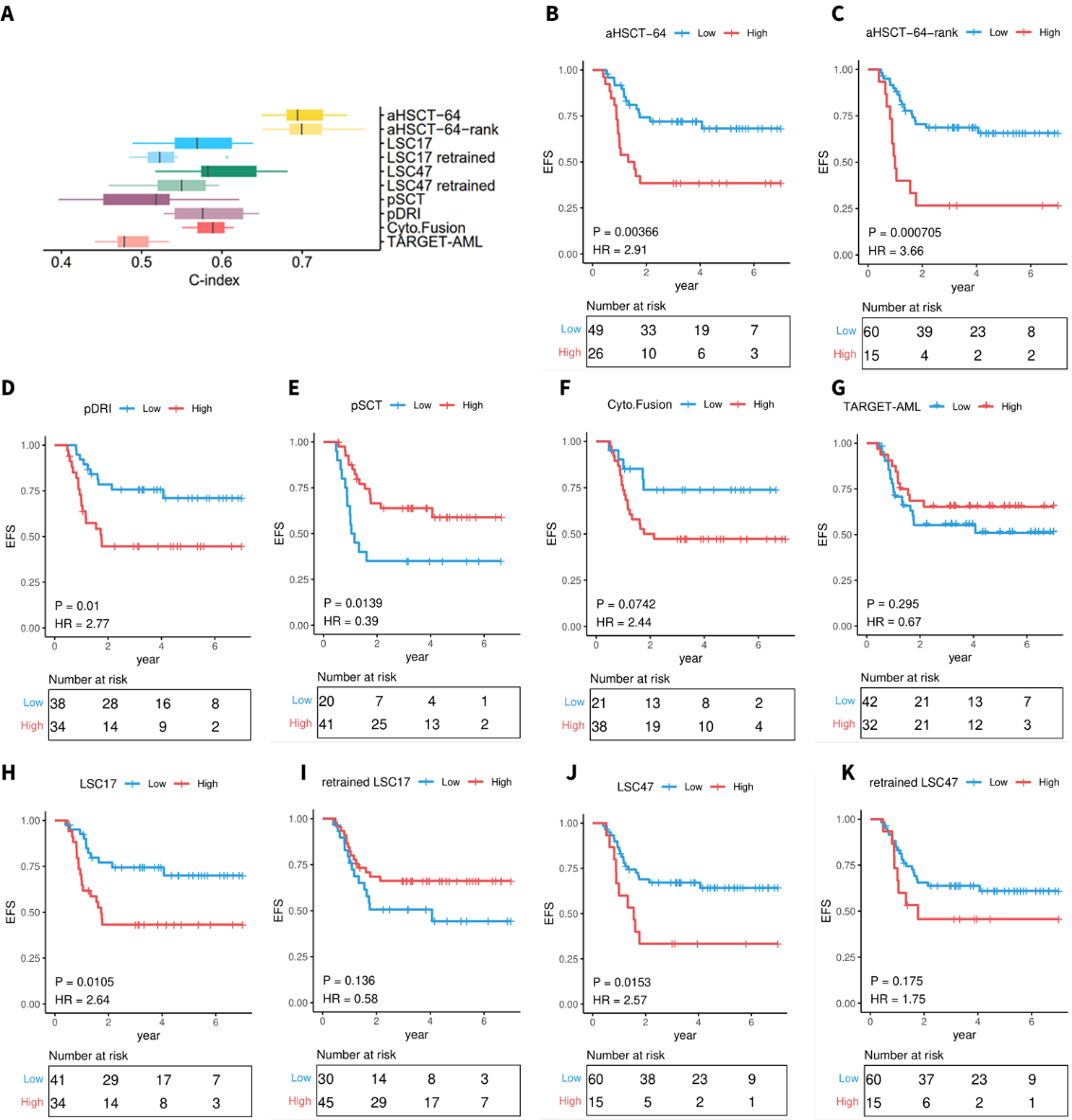


**Figure S5. Evaluation of risk models for event-free survival (EFS) on the discovery allo-HSCT test set.**

(**A**) C-index distribution derived from repeated downsampling model robustness assessment. (**B-K**) Kaplan–Meier analysis comparing EFS between low- and high-risk subgroups stratified by aHSCT-64 (B), rank-based aHSCT-64-rank (C), pDRI (D), pSCT (E), cytogenetic gene fusion-based Cyto.Fusion (F), TARGET-AML risk group (G), leukemia stem cells (LSCs)-based risk model LSC17 (H), LSC17 retained on training sets (I), LSC47 (J), and LSC47 retained on training sets (K).


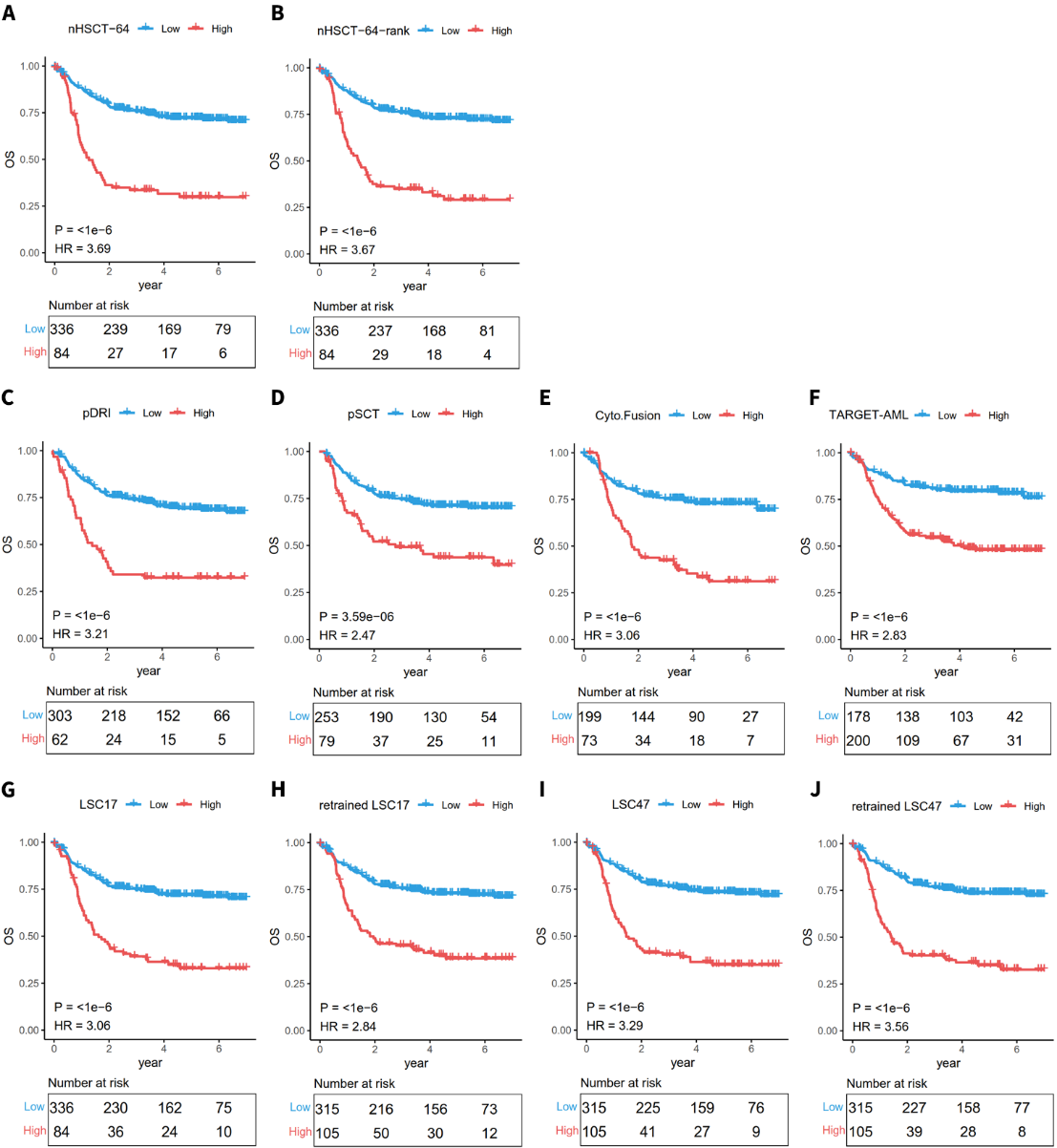


**Figure S6. Evaluation of risk models for overall survival (OS) on the discovery non-HSCT test set.**

(**A-J**) Kaplan–Meier analysis comparing EFS between low- and high-risk subgroups stratified by aHSCT-64 (A), rank-based aHSCT-64-rank (B), pDRI (C), pSCT (D), cytogenetic gene fusion-based Cyto.Fusion (E), TARGET-AML risk group (F), leukemia stem cells (LSCs)-based risk model LSC17 (G), LSC17 retained on training sets (H), LSC47 (I), and LSC47 retained on training sets (J).


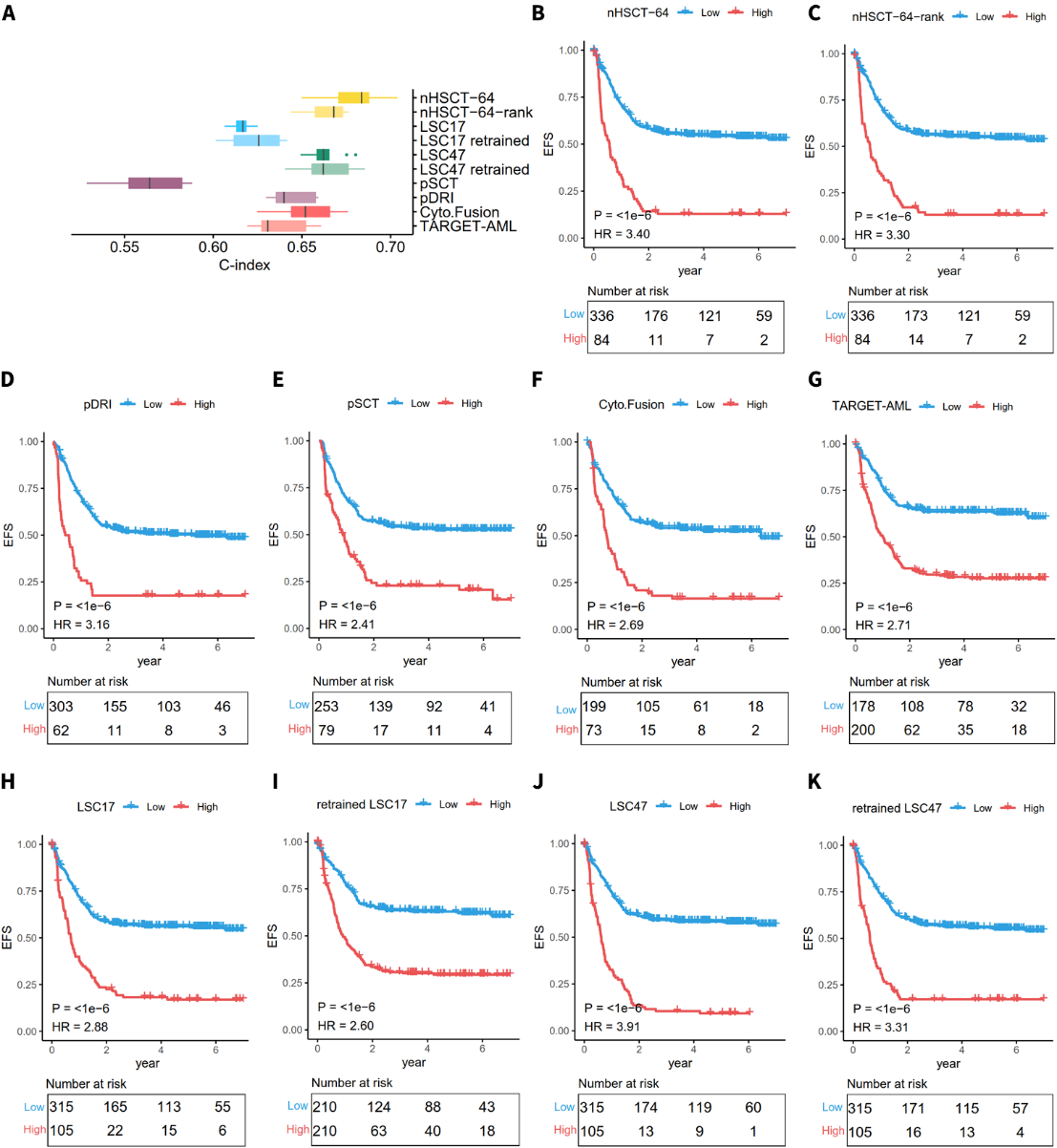


**Figure S7. Evaluation of risk models for event-free survival (EFS) on the discovery non-HSCT test set.**

(**A**) C-index distribution derived from repeated downsampling model robustness assessment. (**B-K**) Kaplan–Meier analysis comparing EFS between low- and high-risk subgroups stratified by aHSCT-64 (B), rank-based aHSCT-64-rank (C), pDRI (D), pSCT (E), cytogenetic gene fusion-based Cyto.Fusion (F), TARGET-AML risk group (G), leukemia stem cells (LSCs)-based risk model LSC17 (H), LSC17 retained on training sets (I), LSC47 (J), and LSC47 retained on training sets (K).


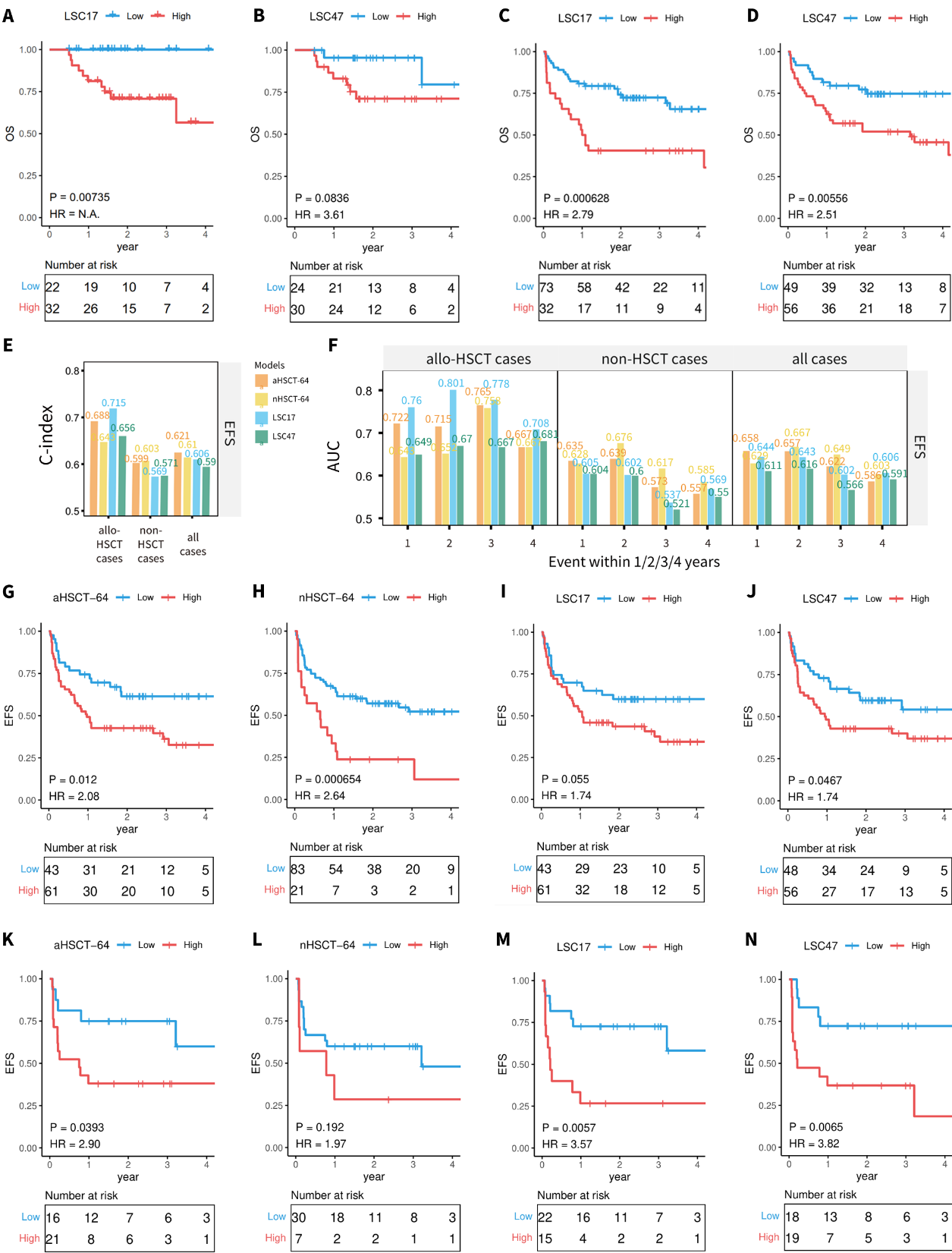


**Figure S8. Validation of HSCT-64 performance in independent cohorts.**

(**A-B**) Kaplan–Meier survival analysis for overall survival (OS) of high- and low-risk subgroups defined by LSC17 (A) and LSC47 (B) in allo-HSCT cases. (**C-D**) Same with (A-B) but in non-HSCT cases. (**E-F**) Comparison of C-index (E) and time-dependent AUC (tAUC) (F) for EFS across different models in allo-HSCT, non-HSCT, and combined cases from independent cohorts. (**G-J**) Kaplan–Meier survival analysis for event-free survival (EFS) of high- and low-risk subgroups defined by aHSCT-64 (H), nHSCT-64 (H), LSC17 (I) and LSC47 (D) in non-HSCT cases. (**K-N**) Same with (G-J) but in allo-HSCT cases.


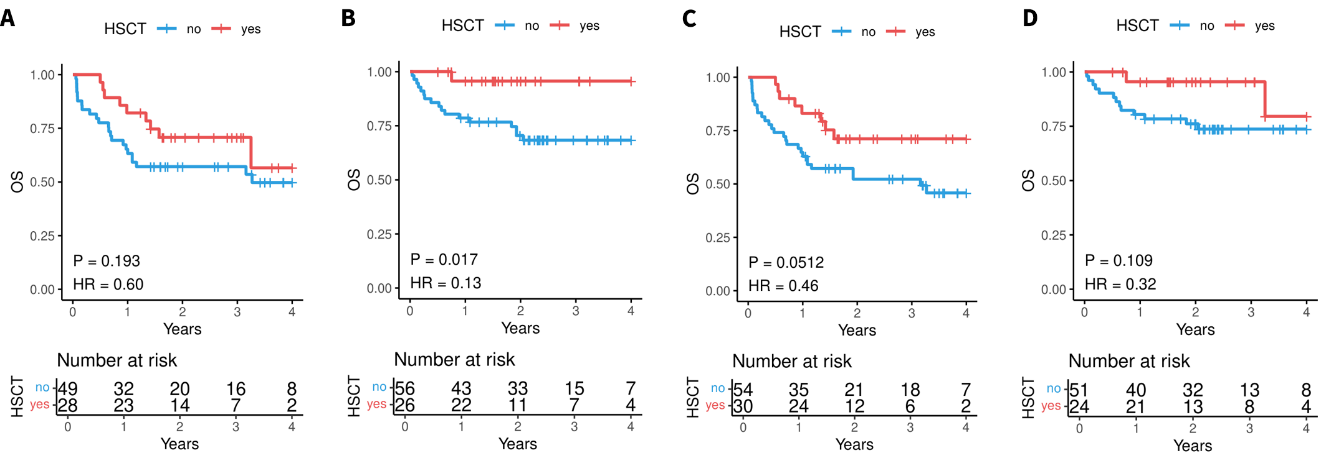


**Figure S9. HSCT decision by single risk model.**

(**A-B**) In high- (A) and low-risk (B) subgroup stratified by single risk model LSC17, KM analysis between allo-HSCT cases and non-HSCT cases. (**C-D**) Same but stratified by LSC47. P, P-value from log-rank test, HR, hazard ratio.


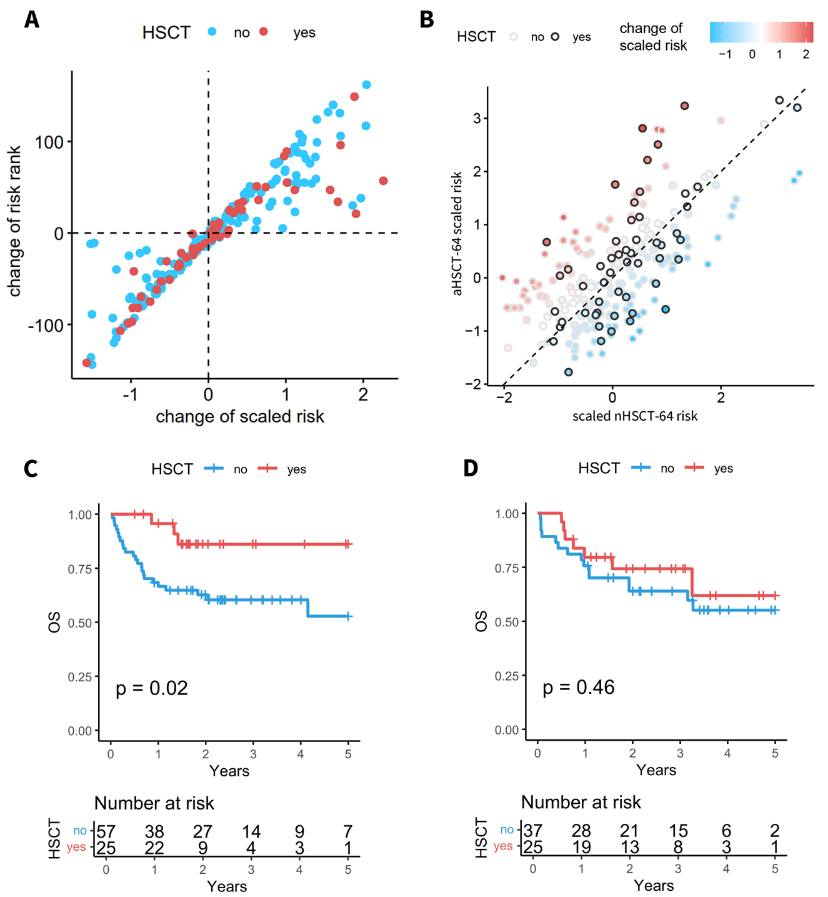


**Figure S10. HSCT decision by parallel-risk model HSCT-64.**

Instead of using risk rank change to identify HSCT-benefiting and HSCT-nonbenefiting subgroups, identification using (scaled) risk change was also assessed. (**A**) relationship between risk rank change and scaled risk change from nHSCT-64 to aHSCT-64. (**B**) Comparison of risk ranks generated by aHSCT-64 and nHSCT-64 models in the independent cohort. Patients with decreased risk rank under aHSCT-64 (blue) were predicted to benefit from HSCT, while those with increased rank (red) were predicted as non-benefiting. (**C**) Among the predicted HSCT-benefiting subgroup, transplanted patients showed significantly better overall survival (OS) than non-transplanted patients. (**D**) In the HSCT-nonbenefiting subgroup, transplantation did not lead to a significant difference in OS.


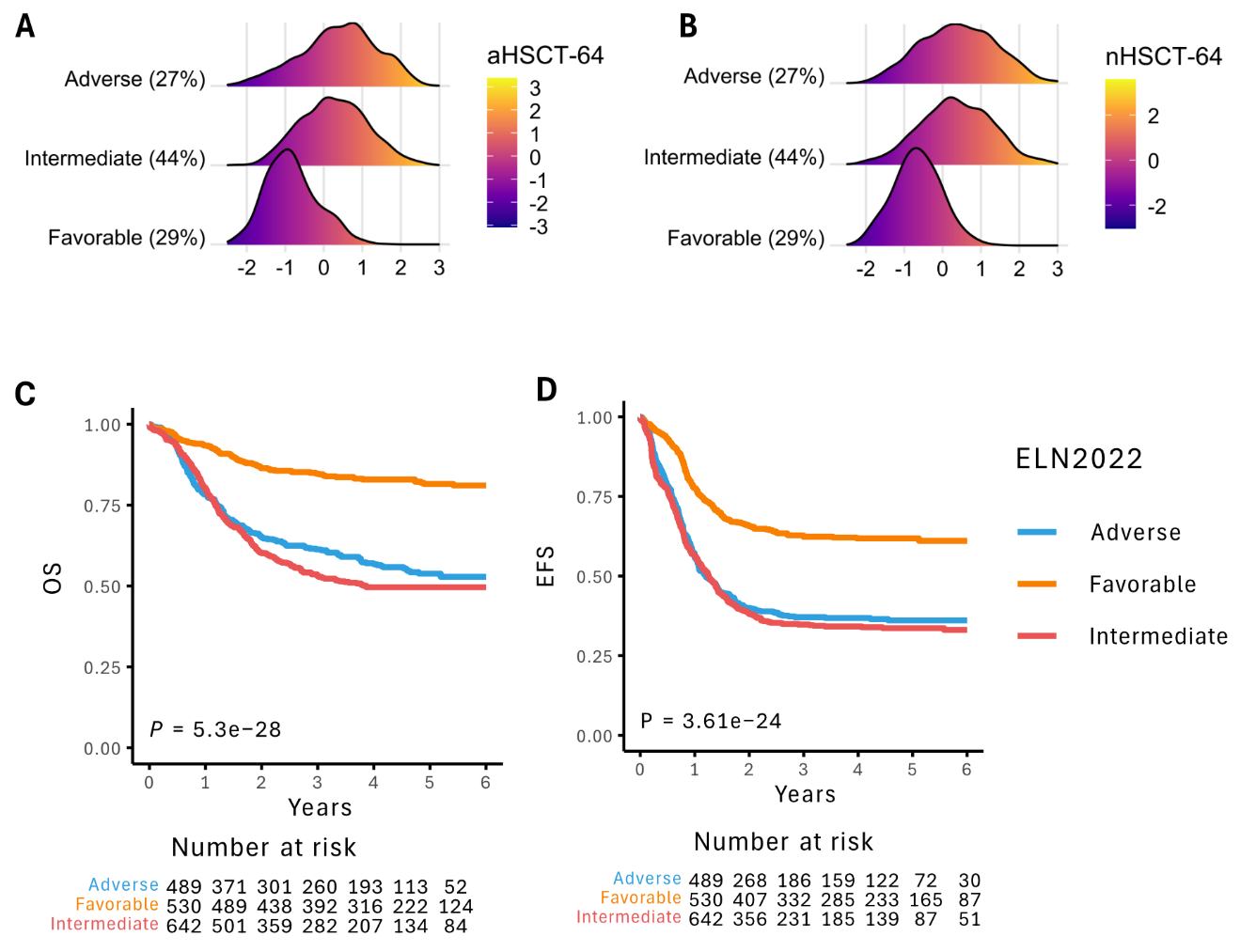


***Figure S11. HSCT-64 risk distribution and clinical outcomes according to the 2022 European Leukemia Net (ELN) risk classification at initial diagnosis.***

*(****A-B****) Distributions of aHSCT-64 (A) and nHSCT-64 (B) risks across ELN subgroups. Patients classified as favorable by ELN exhibited lower risks under both models, whereas those with intermediate and adverse ELN risk showed largely overlapping risk distributions. (****C-D****) Overall survival (C) and event-free survival (D) of ELN subgroups. Consistent with the risk distribution patterns, intermediate and adverse groups demonstrated comparable clinical outcomes.*
