# Supplementary material for: A parallel-risk framework accurately predicts hematopoietic stem cell transplantation outcomes and identifies benefiting patients in pediatric AML
Source: Genes Dis. 2025 Dec 23;13(5):102003. doi: 10.1016/j.gendis.2025.102003 (PMC13273867; doi:10.1016/j.gendis.2025.102003)
Supplement: Multimedia component 3 [file mmc3.docx]

# Supplementary Methods

**Preliminary Gene Feature Selection**

To narrow down the gene set and ensure model robustness in practical applications, genes unsuitable for modeling were excluded. Genes meeting the following criteria were included for further analysis:
(1) protein-coding genes located on autosomal chromosomes;
(2) average (log-transformed) expression across samples ≥ 1;
(3) sufficient variability in (log-transformed) expression levels across samples, with interquartile range (IQR) ≥ log2;
(4) top 1,000 genes were selected based on *p*-values from weighted univariate Cox proportional hazards models with overall survival (OS) weighted by allo-HSCT:non-HSCT = 5:1.

Identification of 64 Core Prognostic Genes

Using the 1,000 selected genes and OS, a multigene weighted ElasticNet-regularized Cox proportional hazards model was fitted to identify prognostic genes with a sample weighting ratio of 5:1 (allo-HSCT:non-HSCT). To reduce the impact of sampling randomness, 70% downsampling was repeated 50 times. The ElasticNet model was repeatedly fitted, and the 64 genes most frequently selected across these 50 repetitions were designated as core prognostic genes.

Selection of the Optimal Prognostic Model

Given the 64 core prognostic genes, predictive models were constructed. To increase model flexibility and software compatibility, survival time was converted into binary classification events for death within 1, 2, 3, 4, 5, and 6 years. This transformation preserved most of the information while avoiding the proportional hazards assumption of the Cox model. Only the 249 allo-HSCT patients were used to train the aHSCT-64 model while 1,398 non-HSCT patients for nHSCT-64 model, with a 7:3 split into training and test sets. This is equivalent to a single model where treatment type (all-HSCT or non-HSCT) interacts with each gene. The following classifiers were trained and evaluated: Ridge-regularized logistic regression, LASSO-regularized logistic regression, ElasticNet logistic regression (0.1 ≤ α ≤ 0.9), random forest, gradient boosting, and support vector machine classifier (SVMC). Model hyperparameters were optimized via 3-fold cross-validation on the training set. To account for the randomness in data splitting and model performance, the train/test split, model training, and evaluation were repeated 10 times with different random number seeds. The optimal model was selected based on the median AUC across the 10 test sets. At last, ridge-regularized logistic regression was selected as final predictive model.

Rank-Based Prognostic Model

Since gene expression quantification from RNA-seq is relative and susceptible to batch effects, using gene expression ranks rather than raw expression values as model inputs can (at least to some extent) address this issue and make the prognostic model more robust.

We refitted the rank-based model using the entire discovery cohorts and applied it to external independent cohorts.

Methodological Rationales

### Why selecting core prognostic genes using the whole discovery cohorts instead of only training cohorts?

In short, this is a tradeoff of data leakage vs. stability of feature selection and model evaluation.

1. Transcriptomic data is high-dimensional and noisy. Selecting stable and reproducible prognostic genes in such settings is difficult when using small subsets (e.g., just training folds). The risk is that different splits will yield different sets of "important" genes due to randomness, not biology. By using the full discovery cohort to select the top 64 prognostic genes, we avoid instability in gene selection due to small size in the training set.
2. The gene set is fixed before model fitting. Only gene selection uses the whole discovery cohort. The model coefficients are still trained on random training/test splits, meaning: there is no circular use of the test labels to fit the model and overfitting from gene selection is minimal.
3. Use of repeated cross-validation (subsampling) to evaluation model performance and avoid overfitting. Since downstream model performance depends on training/testing splitting, evaluating models using repeated subsampling is rather necessary. We perform (1) 50 iterations of subsampling to select stable genes and (2) 10 random train-test splits to evaluate model performance robustly. This repeated-sampling approach ensures that only consistently predictive genes across subsamples are retained — not ones arising from noise or specific data splits.
4. Independent external validation mitigates bias. Any potential optimism introduced by using the full dataset for gene selection is directly tested on completely independent cohorts (CHCMU + SYSMH, *n* = 233). No data leakage flows from discovery to validation. Good performance (C-index = 0.791) supports generalizability.

### Why aHSCT-64 (for allo-HSCT case) and nHSCT-64 (for non-HSCT cases) share the same prognostic genes set?

In short, this depends on the conceptual design of the parallel prognostic modeling framework in HSCT-64.

1. Disease biology is the same but the effect of treatment differs. The transcriptome reflects the underlying leukemia biology at diagnosis, regardless of treatment path. Certain biological pathways (e.g., cell cycle, apoptosis, metabolism) are relevant for both HSCT and non-HSCT outcomes.
2. Facilitates direct, controlled comparison between treatment scenarios. By using the same input features, risk predictions from aHSCT-64 and nHSCT-64 are directly comparable at the individual level. This enables the core idea of the study: for any patient, compare predicted risk under “HSCT” vs “non-HSCT” scenarios. If gene sets differed, such comparison would be confounded by differing input biology — undermining the parallel-scenario simulation.
3. Improves stability and model development feasibility. Selecting different genes for each arm could result in that two models are built on completely different biological signals. Unstable or poorly overlapping gene sets due to sample size limits (especially in the smaller allo-HSCT group). Fixing the gene set makes the modeling task statistically more tractable and less prone to overfitting, especially given limited sample size in HSCT subgroup (n = 249).

### Why reframing the survival regression task as a classification problem to predict death within 1 to 6 years in fitting a/nHSCT-64?

In short, this is a deliberate modeling choice that balances practicality, interpretability, and robustness.

Classification allows use of robust, regularized ML tools

1. Survival data is often right-censored and non-linear. Classical survival models (e.g. Cox) are powerful but assume proportional hazards and may not handle nonlinear interactions or complex gene effects well. In pediatric AML, follow-up durations vary, and event rates are sparse, making accurate time-to-event modeling tricky. Reformulating as classification sidesteps the full censoring model, focusing instead on whether death occurs within fixed time windows (1–6 years), which aligns with clinical interest.
2. Tools like logistic regression (with ridge penalty) or gradient boosting are easier to optimize, have better support in packages like scikit-learn, and are robust to overfitting in high dimensions, unlike Cox models in small datasets. This is particularly advantageous when working with RNA-seq data and small HSCT subgroups (*n* = 249).
3. Proven successful in prior works. The strategy of reframing survival as multi-timepoint classification is increasingly common in bioinformatics, especially in scenarios using omics data, with non-proportional hazards, or aiming to apply models to external cohorts with batch effects. Packages like Multi-task logistic regression for survival analysis (MTLR)[1] [2]and PySurvival[3] adopt this strategy.

Evaluation and Comparison with Existing HSCT Prognostic Models on Internal Test Sets

On the randomly split test sets, existing pediatric AML HSCT prognostic models were evaluated, including the pDRI model, leukemia stem cell-based risk models LSC17 and LSC47, and the previously published pAML SCT model developed by the applicant’s research group (see Table 1). These models were compared based on concordance index (C-index), ROC curves, AUCs, and survival curves for risk stratification in predicting death within 1, 2, 3, 4, 5, and 6 years.

Table 1. Evaluated risk models include clinical-cytogenetic and transcriptomic models.

| **Risk model** | **Publish year** | **Predictive features** | **For pAML** | **For HSCT cases** |
| --- | --- | --- | --- | --- |
| LSC17 | 2016 | Expressions of 17 genes at diagnosis | no | no |
| LSC47 | 2022 | Expressions of 47 genes at diagnosis | no | no |
| pDRI | 2021 | Clinical, cytogenetic, and response indicators after first- and second-phase induction | yes | yes |
| pAML SCT | 2024 | Clinical, cytogenetic, and response indicators after first-phase induction | yes | yes |
| **HSCT-64** | **This article** | Expressions of 64 genes at diagnosis | **yes** | **yes** |

References

1. Haider, H., *MTLR: Survival Prediction with Multi-Task Logistic Regression*. 2019.

2. Yu, C.-N.a.G., Russell and Lin, Hsiu-Chin and Baracos, Vickie, *Learning Patient-Specific Cancer Survival Distributions as a Sequence of Dependent Regressors*, in *NeurIPS Proceedings*. 2011.

3. others, S.F.a., *PySurvival: Open source package for Survival Analysis modeling*. 2019.
